# Van der Waals interactions regulating the hydration of 2-methacryloyloxyethyl phosphorylcholine, the constructing monomer of biocompatible polymers

**DOI:** 10.1038/s41598-022-24841-y

**Published:** 2022-11-27

**Authors:** Masae Takahashi, Sifan Chen, Hiroshi Matsui, Nobuyuki Morimoto, Yuka Ikemoto

**Affiliations:** 1 Graduate School of Science, Tohoku University, Sendai, 980-8578 Japan; 2 Graduate School of Agricultural Science, Tohoku University, Sendai, 980-8572 Japan; 3 Graduate School of Engineering, Tohoku University, Sendai, 980-8579 Japan; 4 Institute for the Promotion of University Function Enhancement, Shimane University, Matsue, 690-8504 Japan; 5 Japan Synchrotron Radiation Research Institute (JASRI)/SPring-8, Sayo, Hyogo 679-5198 Japan

**Keywords:** Physical chemistry, Polymer chemistry, Theoretical chemistry, Chemical physics, Biomaterials, Soft materials, Computational science, Infrared spectroscopy, Soft materials

## Abstract

Van der Waals (VDW) interactions provide fantastic properties for biological systems that function at room temperature. The VDW interaction, which primarily contributes to weak hydrogen bonding, is expected to play a key role in regulating hydrophobic hydration to express the biologically inert biocompatible function of polymerized MPCs (2-methacryloyloxyethyl phosphorylcholine). This report explores at the molecular level the biologically inert function of polymerized MPCs through an array of vibrational spectroscopic and computational characterization of MPC monomers, as temperature-dependent change of intramolecular weak hydrogen bonding. Synchrotron Fourier transform infrared microspectroscopy and terahertz time-domain spectroscopy were used to investigate temperature-dependent spectral changes in the low frequency vibrations of the MPC over the temperature range from cryogenic to room temperature, and the results were analysed by highly reliable well-established density functional theory (DFT) calculations. Complicated spectral features in the low frequency energy region and the uncertain conformations of the MPC in the amorphous powder state are clearly resolved under a polarizable continuum model and dispersion correction to pure DFT calculations.

## Introduction

**Figure 1:**
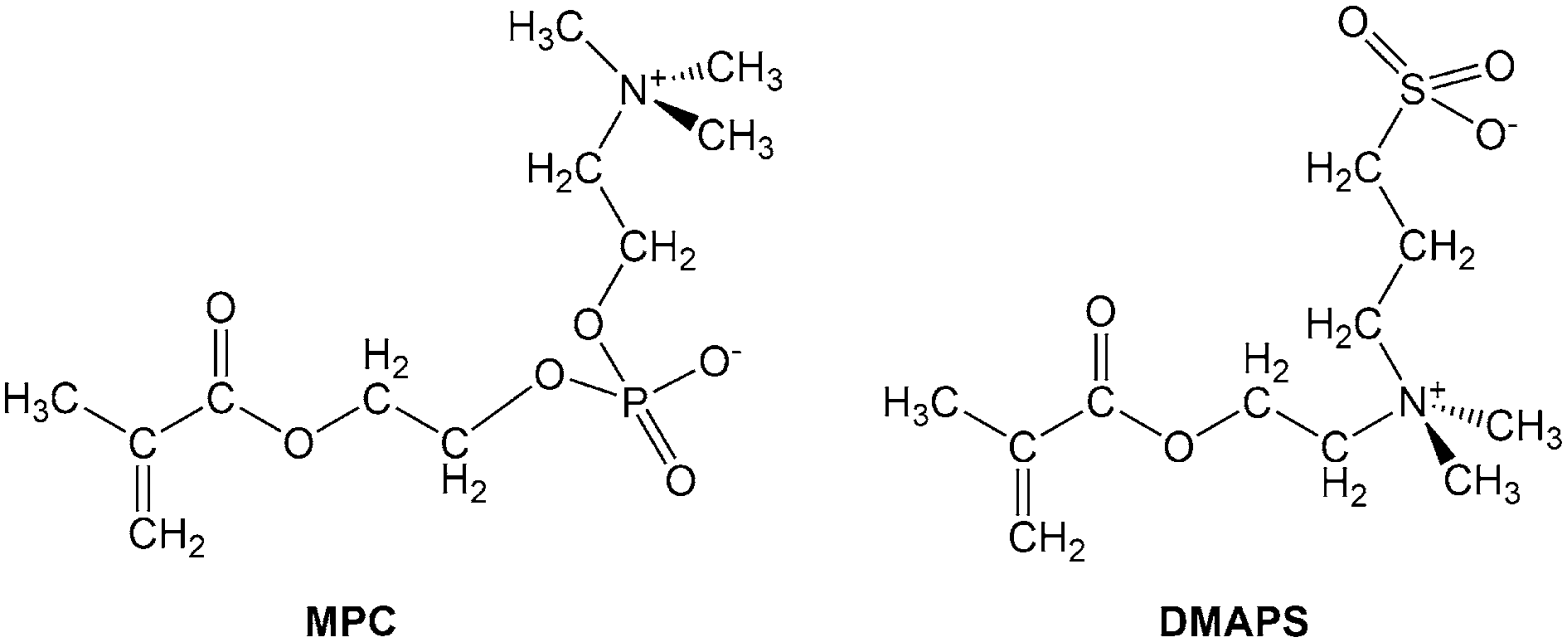
Molecular structures of 2-methacryloyloxyethyl phosphorylcholine (MPC) and 3-[dimethyl-(2-methacryloyloxyethyl) ammonium] propane sulfonate (DMAPS).

Biocompatible materials have promise as artificial materials for biomedical devices. They must have outstanding properties that can resist nonspecific protein adsorption and blood coagulation and can avoid undesired biological reactions such as blood clotting, inflammation, immunoreactions, bacterial adhesion, biofilm formation, cell adhesion, and cell differentiation^1,2^. Despite its importance for applications in biomedical devices, the biocompatible function of these materials is still unclear at the molecular level. One of the most commonly used monomers as a component of biocompatible polymers is 2-methacryloyloxyethyl phosphorylcholine (MPC; Fig. 1), a methacrylate with a phospholipid polar group in the side chain. Based on the molecular structure of phosphatidylcholine in the outer leaflet of eukaryotic plasma membranes, MPC was developed to endow material surfaces with biologically inert functions, such as those possessed by endothelial cells in blood vessels^3^. The biocompatibility and biologically inert function of polymerized MPC is stemmed from the MPC monomer. Several experimental reports^4–6^ suggested that the protein adsorption-resistant properties of the MPC polymer system are closely related to the state of water molecules around the polymer. The unique property of the hydrated MPC polymer is that the degree of hydration increases with increasing temperature in the range from 20 to 50 °C^7^. The mechanism of the temperature-dependent affinity of the MPC polymers for water is still unknown at the molecular level.

The van der Waals (VDW) interaction named in honor of van der Waals, who first introduced attractive interactions between neutral molecules in his equation of state, is a dispersion interaction of pure quantum physical origin^8,9^. It is very weak and easily disturbed by the thermal energy around room temperature. In order to understand fundamentally the mechanism of the temperature-dependent affinity of the MPC polymers for water near room temperature (20–50 °C), we focus on the VDW interaction in MPC monomers. The VDW interaction has been found to be a constructing force of promising materials such as the VDW heterostructure^10–13^, quantum liquid^14,15^, the VDW bonded magnet^16^, molecular diode^17^, and Rydberg gas^18^. We recently used terahertz (THz) and far-infrared (FIR) spectroscopies to detect the effects of weak VDW forces that control the environmental sensitivity of a biocompatible material, 3-[dimethyl-(2-methacryloyloxyethyl) ammonium] propane sulfonate (DMAPS; Fig. 1)^19^. The effect of VDW forces manifests itself as the formation of weak hydrogen bonds at the VDW limit. The weak hydrogen bond is one of three classes of hydrogen bonds ranging from weak (VDW limit) to strong (covalent-bond limit) depending on its strength^20^, and the dispersion force, one of three interactions (electrostatic, induction, and dispersion) that contribute to hydrogen bonding, primarily contributes to weak hydrogen bonds at the VDW limit.

Low-frequency vibrations in the THz and FIR regions are very sensitive to the local environment in which the molecule resides^21^. Over the last two decades, THz spectroscopy has become an increasingly popular technique^22–24^ and has probed the properties of a vast range of materials and compounds^19,21,25–38^. Due to advances in theoretical methods, the effect of the environment of each molecule in a crystal on low-frequency vibrations has been examined very accurately using solid-state first-principles calculations^21,26^. However, most biological materials are not crystalline when they function in vivo. In our previous paper^19^, by incorporating the effects of surrounding molecules into the calculation with a polarizable continuum model, we have successfully resolved the complicated spectral features in the low-frequency energy region and the uncertain conformations of materials in the amorphous powder state. Our previous paper^19^ revealed that the main cause of the experimentally observed temperature-dependent spectral changes is the strength change or formation/cleavage of intramolecular weak hydrogen bonds at the VDW limit. It was found in the paper^19^ that thermo-responsiveness is detected mainly in two frequency regions, the torsional mode of the methyl group on the nitrogen atom (FIR) and the torsional mode of the side chain (THz).

**Figure 2:**
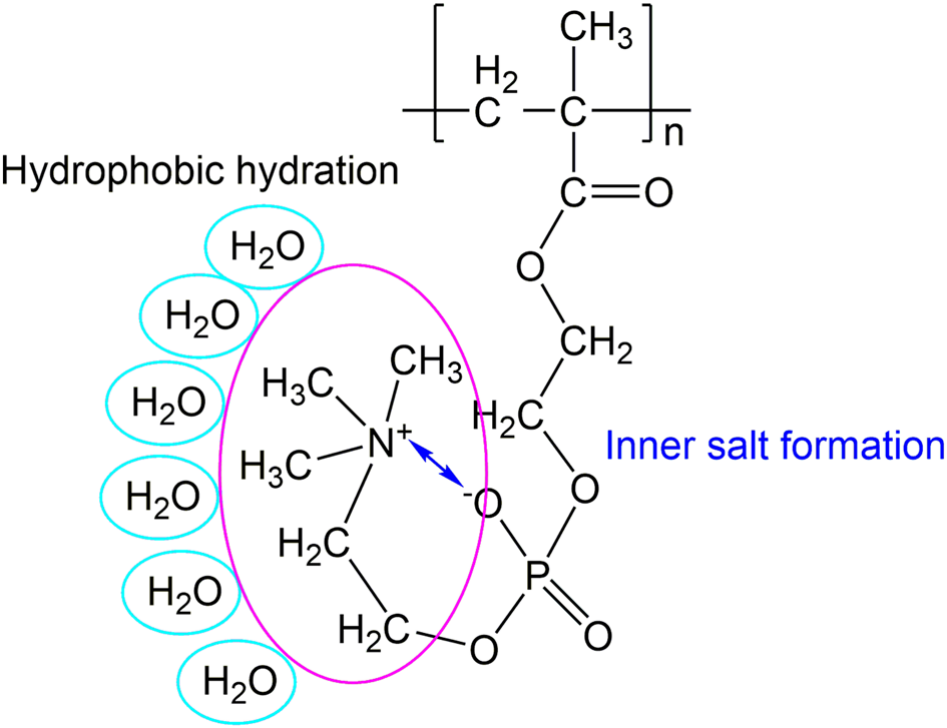
Hydrophobic hydration of polymerized MPC under inner salt formation indicated by blue arrow.

MPC has a trimethyl ammonium mono-cation at the terminal, while DMAPS has a dimethyl ammonium mono-cation in the middle (Fig. 1). The methacrylate group of MPC at one terminal is the polymerizable unit, and the polar phosphorylcholine group at the other terminal forms the inner salt (Fig. 2). Three methyl groups on positive nitrogen form favorable interactions with water through hydrophobic hydration^39^. The temperature dependence of the low-frequency spectrum is predicted to differ between MPC and DMAPS due to differences in the position of cationic ammonium and thus in weak intramolecular hydrogen bonds. This report explores at the molecular level the biologically inert function of the polymerized MPC through an array of vibrational spectroscopic and computational characterization of MPC monomers, as temperature-dependent change of weak hydrogen bonds. Synchrotron Fourier transform infrared (FTIR) microspectroscopy and THz time-domain spectroscopy are used to investigate temperature-dependent spectral changes in the low frequency vibrations over the temperature range from cryogenic to room temperature, and the results are analyzed by highly reliable well-established density functional theory (DFT) calculations. We focus on two frequency regions in which significant spectral changes were observed in our previous study^19^ on DMAPS, a constructing monomer for biocompatible polymeric materials. We also focus on the temperature range from cryogenic to near room temperature, because cleavage of weak hydrogen bonds at the VDW limit occurs at approximately 100–150 K^19^. We perform a gas-phase calculation instead of a solid-state calculation for peak assignment because the sample studied here is fully amorphous. We incorporate here the dielectric constant into the gas-phase calculation as the effect of the surrounding molecules with a large dipole moment.

## Results and discussion

### FIR spectra and their vibrational modes

**Figure 3:**
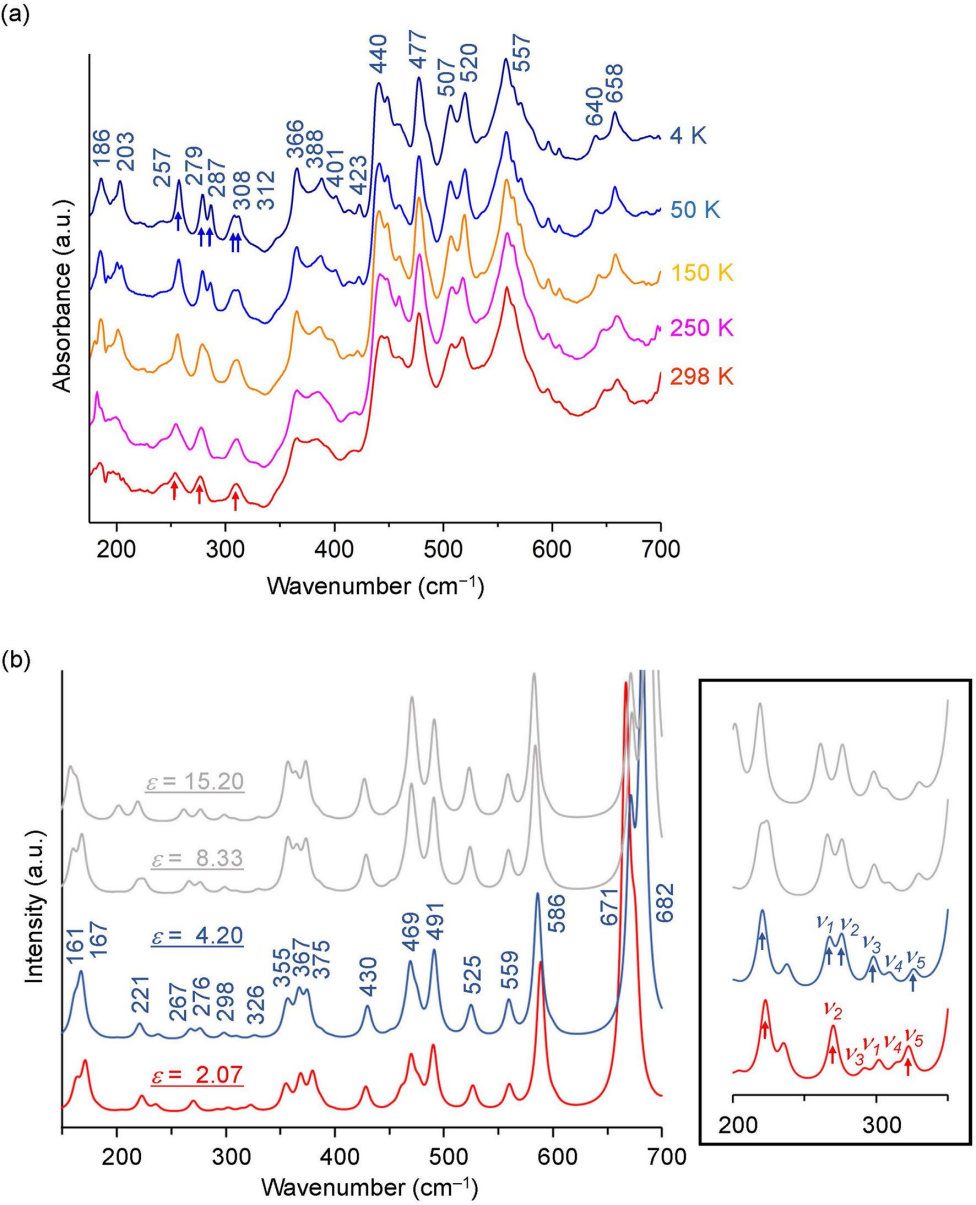
Experimental and calculated FIR spectra of MPC. Both of these spectra are vertically offset. (**a**) Temperature-dependent experimental spectra. The frequency of each peak at 4 K is given in cm^−1^. Five peaks at 4 K and three peaks at 298 K observed in the frequency range of 250–350 cm^−1^ are indicated by blue and red arrows, respectively. (**b**) Calculated spectra with four dielectric constants for a MPC conformation that provides the best-fitting FIR spectra. The frequencies at *ε* (dielectric constant) = 4.20 are given in cm^−1^. The full width at half maximum (FWHM) is set to the same value as that of the well-separated sharp peak at 423 cm^−1^ experimentally observed at 4 K (FWHM = 4.4 cm^−1^). A magnification of 200–350 cm^−1^ is shown on the right. Five modes located between 250 and 350 cm^−1^ are denoted as *ν*_*1*_–*ν*_*5*_. Peaks indicated by blue and red arrows correspond to five peaks observed at 4 K and three peaks observed at 298 K, respectively.

The FIR spectral profile of MPC was measured by synchrotron FTIR microspectroscopy to investigate the behaviour at low temperature where VDW interactions become effective. A reversible temperature-dependent spectral change was obtained between the cooling process from room temperature to 4 K and the heating process from 4 K to room temperature. Although the MPC is highly hygroscopic, the absence of strong peaks in water libration mode at approximately 600 cm^−1^ indicates that the MPC under measurement is sufficiently dry and free of water. No standard definition of the energy region currently differentiates FIR from THz. For convenience, this text describes the spectrum measured with a THz time-domain spectrometer as the THz spectrum and the spectrum measured with an FTIR spectrometer as the FIR spectrum. Figure 3a shows the MPC’s temperature-dependent FIR spectra. The overall spectral profile hardly changes upon cooling from room temperature to 4 K, except for peak sharpening and a slight peak shift. In more detail, peaks below 350 cm^−1^ show significant temperature dependence, such as peak splitting by cooling, whereas peaks above 350 cm^−1^ show no noticeable temperature dependence on the number of peaks. Three peaks were observed between 250 and 350 cm^−1^ at 298 and 250 K, as indicated by the red arrows. At low temperatures, peaks split into two peaks near 280 and 310 cm^−1^, and five peaks were totally observed between 250 and 350 cm^−1^, as indicated by the blue arrows. The phenomenon of peak splitting at approximately 300 cm^−1^ due to cooling has also been previously reported for the amorphous biocompatible monomer DMAPS^19^. It is well known that conformational differences are distinguished from each other in the FIR frequency region. In fact, the various conformations for MPC showed quite different spectral patterns in our DFT calculations. Therefore, no remarkable spectral change from room temperature to 4 K suggests no conformational change due to cooling. In addition, the possibility of a conformational mixture or a dimer formation is excluded due to the number and sharpness of the measured peaks in the frequency region below 350 cm^−1^, although the conformation in the amorphous powder of MPC is unknown.

Figure 3b shows the FIR spectrum calculated with four dielectric constants for the MPC conformation that gives the best-fitting FIR spectrum. In the frequency range above 350 cm^−1^, no significant differences, such as peak splitting, were observed between the spectra of different solvents, although the relative intensities of some peaks changed and the frequency values of some peaks shifted slightly. On the other hand, below 350 cm^−1^ where a temperature-dependent spectral change was experimentally observed, significant differences were observed between the spectra of different solvents. The right panel of Fig. 3b gives a magnification in the range between 200 and 350 cm^−1^. Five modes *ν*_*1*_–*ν*_*5*_ located in the frequency region of 250–350 cm^−1^ showed remarkable changes with changes in dielectric constant. No peak splitting was observed near 280 cm^−1^ in the spectrum calculated at *ε* = 2.07, while two peaks were observed near 280 cm^−1^ in the three spectra calculated at *ε*  = 4.20, 8.33, and 15.20. Experimentally, one peak near 280 cm^−1^ was observed at 298 and 250 K to split into two peaks by cooling. Therefore, the spectrum calculated at *ε* = 2.07 appears to correspond to the experimental spectra at 298 and 250 K. At the experimental peak near 257 cm^−1^, which corresponds to the calculated peak near 220 cm^−1^, no significant temperature dependence was observed. Therefore, the spectrum at *ε* = 4.20, where the peak near 220 cm^−1^ is most similar to that at *ε* = 2.07, would be the most likely spectrum corresponding to the experimental spectrum at low temperature. The frequency values corresponding to the 18 distinct peaks in the experimental spectrum at 4 K are shown in the spectrum at *ε* = 4.20 in Fig. 3b. The maximum discrepancy is 39 cm^−1^ between the calculated frequency at *ε* = 4.20 and the experimental frequency at 4 K. The three experimentally observed peaks indicated by the red arrows in Fig. 3a correspond to the calculated three peaks indicated by the red arrows in the right panel of Fig. 3b (*ε* = 2.07). The five experimentally observed peaks indicated by the blue arrows in Fig. 3a correspond to the calculated five peaks indicated by the blue arrows in the right panel of Fig. 3b (*ε* = 4.20). It is found that the new peaks that appear at low temperatures in the experiment are modes *ν*_*1*_ and *ν*_*3*_.

The dielectric constants of MPC (*ε* = 2.07, 4.20) are lower than those of DMAPS^19^ (*ε* = 8.33, 15.20). In addition, the tendency for a high dielectric constant to correspond to low temperature states and for a low dielectric constant to correspond to high temperature states are the opposites of the tendency observed with DMAPS^19^. One probable interpretation is that for polar molecules with a permanent dipole moment such as DMAPS and MPC, the mobility to allow orientation of molecules is related to the temperature dependence of the dielectric constant in the external applied field. That is, the phenomenon of the dielectric constant increasing at high temperatures could be due to the high mobility at high temperatures that facilitates the molecular orientation in the external field like the difference in dielectric constant between water and ice. The high mobility at high temperatures facilitates the molecular orientation in the external applied field, while it randomizes the molecular orientation in the absence of the external field. The phenomenon that the dielectric constant decreases at high temperature could be due to the low mobility that inhibits the orientation of molecules in the external applied field and the randomness of orientation of the molecular dipoles enhanced by thermal energy at high temperatures. Different temperature dependence of the dielectric constant suggests that MPC is less mobile than DMAPS.

**Figure 4:**
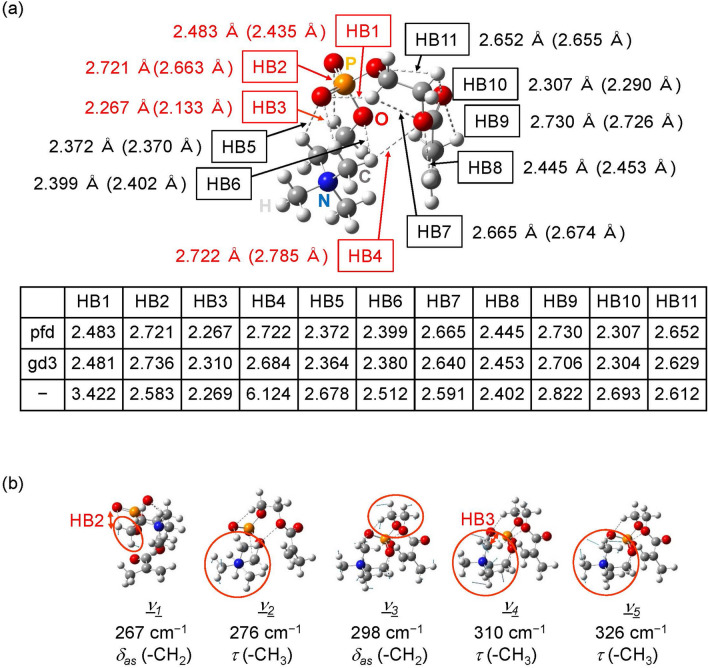
(**a**) Intramolecular hydrogen bond distances of the most likely conformer of MPC calculated at a dielectric constant of 4.20. Intramolecular hydrogen bonds, HB1–HB11, are indicated by the dashed lines. The bond lengths at a dielectric constant of 2.07 are given in parentheses. The four hydrogen bonds that show a remarkable change in length due to the difference in dielectric constant are marked in red. The table shows the optimized bond lengths in Å calculated at the dielectric constant of 4.20 with the Petersson-Frisch dispersion model (pfd)^40^, the D3 version of Grimme’s dispersion (gd3)^41^, and without dispersion correction (–). (**b**) Vibrational modes given as *ν*_*1*_–*ν*_*5*_ in the right panel in Fig. 3b (*δ*_*as*_: asymmetric bending in plane or rocking, *τ*: torsion). The frequency of each mode is the result calculated with a dielectric constant of 4.20 using the pfd model for dispersion correction. Intramolecular hydrogen bonds are indicated by the dashed lines. The blue arrows indicate the atomic displacement vectors. Mainly moving parts are marked with red circles. The stretching vibration of an intramolecular hydrogen bond is indicated by a red arrow along with the name of hydrogen bond.

Figure 4a shows the intramolecular hydrogen bond distances of the most likely conformer. The eleven intramolecular CH…O distances are calculated to be less than 2.77 Å (the limit of VDW contacts for weak hydrogen bonds at the same level of calculations^19^) at a dielectric constant of 4.20, which suggests the formation of weak hydrogen bonds. The bond lengths of HB1–HB11 calculated with different dispersion corrections are found to be the same within a difference of 0.04 Å (table in Fig. 4a). The bond length larger than 2.77 Å for HB1, HB4, and HB9 calculated without dispersion correction suggests that the dispersion force mainly contributes to the hydrogen bonds of HB1, HB4, and HB9, stabilizing the conformation. When comparing the intramolecular hydrogen bond distances between the results calculated with dielectric constants of 4.20 and 2.07, the four intramolecular hydrogen bonds HB1–HB4 marked in red showed remarkable changes in distance. At high dielectric constant corresponding to low temperatures, the distances of HB1–HB3 are significantly elongated, and the distance of HB4 is shortened. The distance of HB4 at *ε* = 2.07 that corresponds to high temperatures is longer than 2.77 Å, suggesting that a bond break occurs. The opposite change with a change of dielectric constant in distance of HB4 to that of HB1–HB3 can be interpreted by a change in N^+^–O^−^ distance. Changes in dielectric constant affect the electrostatic charge-charge interaction between negative oxygen on phosphorus and positive nitrogen, causing a change in N^+^–O^−^ distance. The N^+^–O^−^ distance *r*(N^+^ − O^−^) in a low dielectric constant environment is shorter than in a high dielectric constant environment: *r*(N^+^–O^−^) is 3.581Å for *ε* = 2.07 and 3.658 Å for *ε* = 4.20. HB3 is a bond between the negative oxygen of N^+^–O^−^ and the hydrogen of the methyl group on the positive nitrogen of N^+^–O^−^, and shows the largest shrinkage of the three hydrogen bonds (HB1–HB3) by lowering the dielectric constant. HB3 and HB4 are bonds formed by different hydrogens of the same methyl group on positive nitrogen of N^+^–O^−^, each connecting to different oxygen in opposite directions. Therefore, as the HB3 distance decreases, the HB4 distance increases. The four hydrogen bonds HB1–HB4 are not connected to the methacryloyl group used for polymerization (Fig. 2), and thus, their binding is not directly inhibited by the polymerization. When made into polymers, steric hindrance may limit the movement of functional groups, but changes in the bond distance of HB1–HB4 would occur as with monomers.

Figure 4b shows the five vibrational modes given as *ν*_*1*_–*ν*_*5*_ in the right panel in Fig. 3b, calculated with a dielectric constant of 4.20. There is no significant difference in the assigned modes *ν*_*1*_–*ν*_*5*_ between different dielectric constants, 2.07 and 4.20. Three of the five peaks, *ν*_*2*_, *ν*_*4*_, and *ν*_*5*_, are assigned to the torsion mode of the methyl group (*τ* (–CH_3_)) on positive nitrogen. The remaining two of the five, *ν*_*1*_ and *ν*_*3*_, which appeared in experiments at low temperatures, are assigned to the methylene rocking mode (*δ*_*as*_ (–CH_2_)). This is different from DMAPS, where cooling causes peak splitting for the torsion mode of the methyl groups on nitrogen. Since these five modes contain little or no movement of the nitrogen atom itself and the oxygen atoms of the PO_4_ moiety, the frequencies in these modes are presumed to be largely unaffected by changes in the electrostatic interaction between N^+^ and O^−^ due to changes in dielectric constant. The vibrational frequency shifts to reflect changes in both force fields and mass distribution. The formation of weak hydrogen bonds impedes the torsional and/or rocking motion and increases the frequency value. If the stretching vibrations of the intramolecular hydrogen bonds are mixed with the torsional and/or rocking modes, the elongation of the hydrogen bond distances weakens the hydrogen bond interaction and causes a redshift. Modes *ν*_*1*_ and *ν*_*4*_ contain stretching vibrations of HB2 and HB3, respectively, as indicated by the red arrows in Fig. 4b. As shown in Fig. 4a, the bond lengths of HB2 and HB3 are longer at *ε* = 4.20 than at *ε* = 2.07. Therefore, as observed in the right panel in Fig. 3b, the frequencies of modes *ν*_*1*_ and *ν*_*4*_ are lower for *ε* = 4.20 than for *ε* = 2.07. The peak splitting observed at low temperature seems to be due to the appearance of an enhanced and low-frequency-shifted *ν*_*1*_ next to *ν*_*2*_ at *ε* = 4.20, corresponding to the low temperature state. Five vibrational modes *ν*_*1*_–*ν*_*5*_ are local intramolecular vibrations. These modes contain no or little movement of the methacryloyl group used for polymerization. The temperature-dependent spectral changes of the peaks assigned to *ν*_*1*_–*ν*_*5*_ are expected to occur in the same way as monomers even when polymerized, although the frequency may increase due to steric hindrance caused by polymerization.

### THz spectra and their vibrational modes

**Figure 5:**
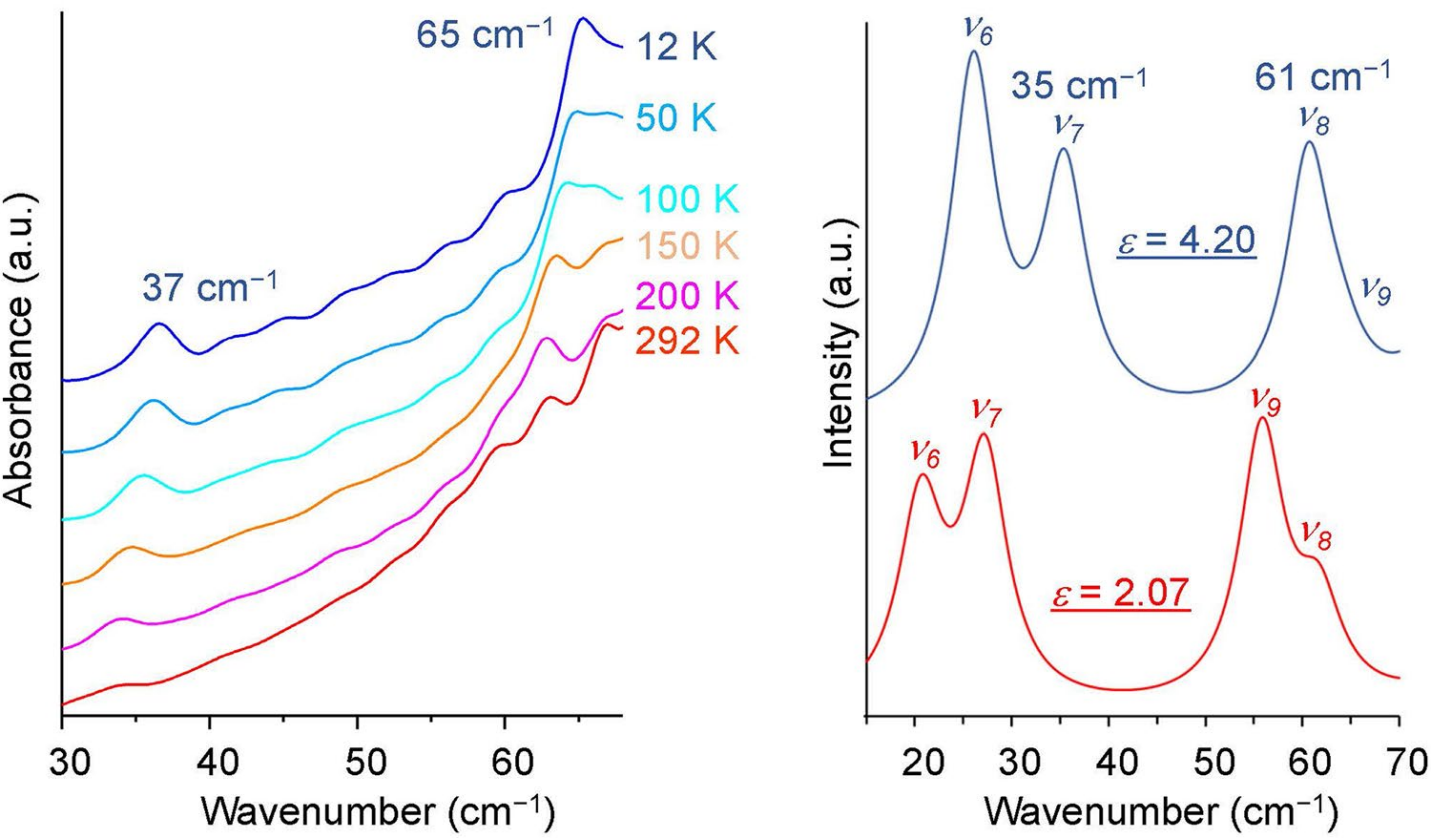
Experimental (left) and calculated (right) THz spectra of MPC. Both of these spectra are vertically offset. The FWHM of calculated spectra is set to the same value as that of the experimental peak at 37 cm^−1^ at 12 K (FWHM = 2.8 cm^−1^). The four modes obtained in the calculations are denoted as *ν*_*6*_–*ν*_*9*_.

Figure 5 shows the experimental and calculated THz spectra of MPC. As concluded in the FIR spectra, the calculated spectra with dielectric constants of 4.20 and 2.07 (blue and red lines in the right panel of Fig. 5, respectively) correspond to the low- and high-temperature spectra, respectively. The monotonically growing background from low to high frequencies observed in the experimental spectra (left panel of Fig. 5) is the characteristics of the amorphous THz spectra^42^. Unlike the THz spectrum of polymers^43^, the MPCs studied here are monomer molecules, thus excluding the possibility of partial crystallization as in amorphous one-dimensional long polymer chains. In the experimental spectra (left panel of Fig. 5), two peaks were observed, which were simply blueshifted with cooling. In the spectra calculated with *ε* = 4.20 and 2.07 (right panel of Fig. 5), there were four modes *ν*_*6*_–*ν*_*9*_ below 70 cm^−1^. Considering the intensity and the experimentally detectable region, the two peaks observed in the 12 K experimental spectrum would be assigned to the two modes *ν*_*7*_ (35 cm^−1^) and *ν*_*8*_ (61 cm^−1^) calculated at *ε* = 4.20. Similarly, the two peaks observed in the experimental spectrum at higher temperatures would be assigned to the two modes *ν*_*7*_ and *ν*_*9*_ calculated at *ε* = 2.07. The frequencies of the two modes *ν*_*7*_ and *ν*_*8*_ calculated at *ε* = 4.20 are on the higher frequency side than the two modes *ν*_*7*_ and *ν*_*9*_ calculated at *ε* = 2.07. The change in dielectric constant would be one possibility to explain the experimentally observed blueshift by cooling. The temperature-dependent frequency shift in the vibrational spectrum of an amorphous sample has not been fully elucidated. In the crystalline system, it is well established that possible causes of the frequency shift with temperature are thermal expansion of crystal and phonon-mediated scattering processes (modification of electronic excitation by coupling to lattice vibrations (phonons))^44^. Furthermore, in weakly bonded crystalline systems, phonon-mediated scattering processes are major cause, and the effect of the changes in cell volume with temperature is negligible^25^. Lattice vibrations (phonons) of periodic crystals do not exist in amorphous systems, eliminating the possibility of phonon-mediated scattering processes. In addition, with respect to thermal expansion, the effects of temperature-dependent changes due to surrounding molecules in randomly and inefficiently packed amorphous systems would be even more negligible than in efficiently and orderly packed weakly bonded crystalline system.

**Figure 6:**
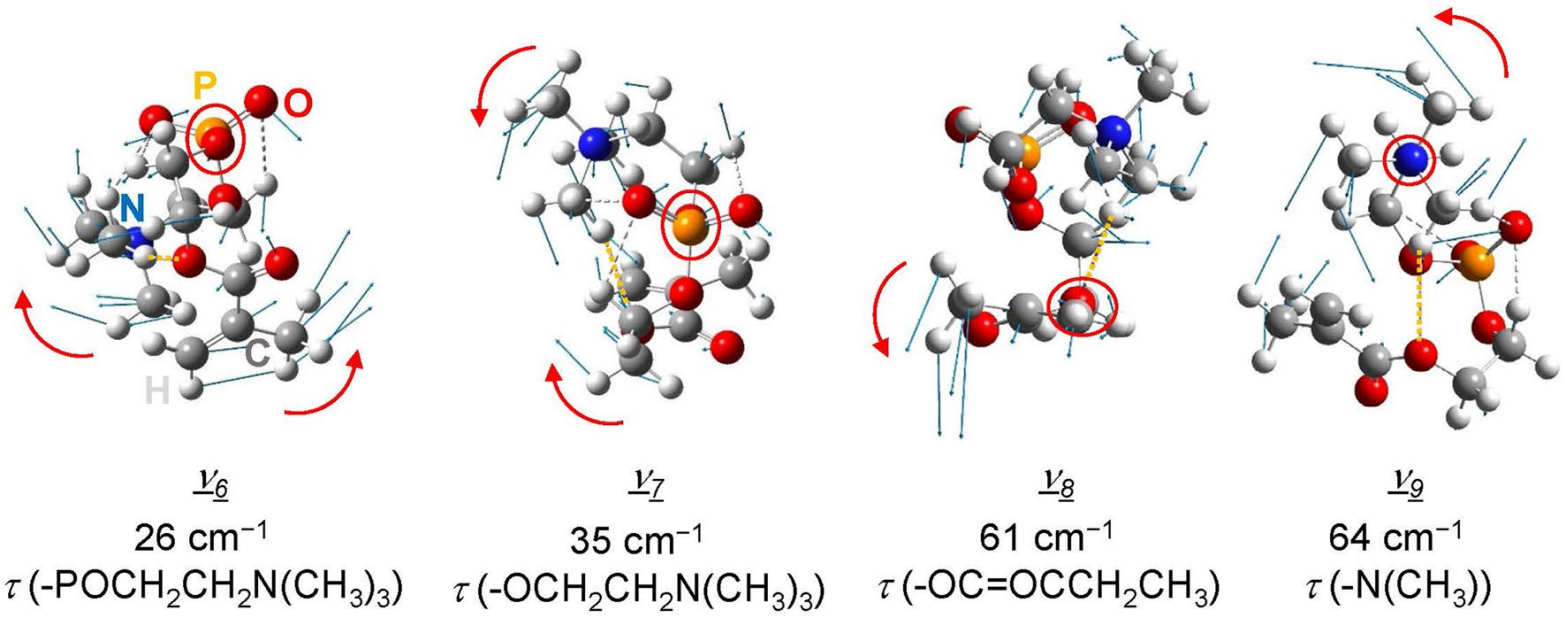
Vibrational modes given as *ν*_*6*_–*ν*_*9*_ in Fig. 5 (*τ*: torsion). The frequency of each mode is the result calculated with a dielectric constant of 4.20. The blue arrows indicate the atomic displacement vectors. Intramolecular hydrogen bonds are indicated by the dashed lines, and the orange dashed line is the hydrogen bond HB4 given in Fig. 4a. The red circle represents the two atoms that form the torsion axis, and the movement around the torsion axis is indicated by the red arrows.

Figure 6 shows the four vibrational modes calculated with a dielectric constant of 4.20, which are given as *ν*_*6*_–*ν*_*9*_ in Fig. 5. There is no significant difference in the assigned modes *ν*_*6*_–*ν*_*9*_ between different dielectric constants, 2.07 and 4.20. All four peaks obtained below 70 cm^−1^ are assigned to the side-chain torsion mode. Since these modes contain little or no N^+^–O^−^ stretching, the frequencies in these modes are presumed to be largely unaffected by changes in the electrostatic interaction between N^+^ and O^−^ due to changes in dielectric constant. Modes *ν*_*6*_, *ν*_*7*_, and *ν*_*9*_ contain stretching vibrations of HB4 indicted by the orange dashed line in Fig. 6, while mode *ν*_*8*_ does not contain any stretching vibrations of intramolecular hydrogen bonds. From the bond length of HB4 in Fig. 4a, HB4 is cleaved at a dielectric constant of 2.07 corresponding to high temperatures. Therefore, for *ε* = 2.07, where HB4 is cleaved, the frequencies of *ν*_*6*_, *ν*_*7*_, and *ν*_9_ are lower than those of *ε* = 4.20, and the frequency of *ν*_*8*_ changes scarcely between the results of *ε* = 4.20 and 2.07. In any of the four side-chain torsion modes *ν*_*6*_–*ν*_*9*_, the two atoms that form the torsion axis are not included in the methacryloyl group used for polymerization. Temperature-dependent spectral changes at the peaks assigned to *ν*_*6*_–*ν*_*9*_ are expected to occur in the same manner as monomers even when polymerized, although the frequency may change due to steric hindrance or fixation of one terminal caused by polymerization.

### Conformation of MPC

**Figure 7:**
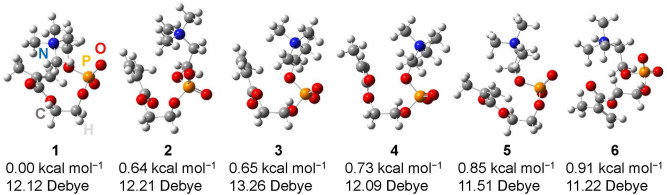
Six most stable conformers **1**–**6** common to solvents of *ε* = 8.33 and 15.20 at the B3LYP/6-311++G(d,p) level. The relative energies from conformer **1** and the dipole moments of each conformer are the results with dielectric constant *ε* = 15.20 and without dielectric constant, respectively, at the B3LYP/6-311++G(d,p) level.

We investigated all possible conformations of MPC molecules to elucidate the conformation of MPC in the amorphous state studied here. First, we determined the stable structure of two terminals, the phosphorylcholine and methacryloyl moieties. For the phosphorylcholine terminal, we obtained two forms, boat-shaped and chair-shaped, which connect positive nitrogen and negative oxygen to form a six-membered ring of O^–^–P–O–C–C–N^+^. The *all-trans* linear form without the N^+^–O^−^ connection was not obtained as a stable minimum. The boat form is 1.1 kcal mol^−1^ more stable than the chair form at the B3LYP/cc-pVTZ level with zero-point energy corrections. For the methacryloyl terminal, we obtained two forms of transoid and cisoid for the two double bonds of C=O and C=CH_2_. The transoid is only 0.3 kcal mol^−1^ more stable than the cisoid at the B3LYP/cc-pVTZ level with zero-point energy corrections. Next, we searched for all possible conformations of the middle O–C–C–O moiety with the methacryloyl moiety of transoid –C(=O)–C(CH_3_)=CH_2_ and the phosphorylcholine moiety of the boat-form O^–^–P–O–C–C–N^+^ ring. We obtained 48 conformations, starting from 81 conformations in the middle O–C–C–O part. We then investigated the effect of the solvent *ε* = 8.33 and 15.20 on the conformational stability. Dielectric constants *ε* = 8.33 and 15.20 reproduced well the FIR and THz spectra of DMAPS in our previous paper^19^. The effect of lower dielectric constant is estimated to be between the results of *ε* = 8.33 and without solvent. The six most stable conformations, **1**–**6**, shown in Fig. 7, are common to the solvents *ε* = 8.33 and 15.20 at the B3LYP/6-311++G(d,p) level, but in a different order of stability. These six conformers are included in the stable conformations within an energy difference of no more than 5 kcal mol^–^^1^ from the most stable conformers in solvent-free calculations. The most stable conformation (**1**) is the same with and without solvent (*ε* = 8.33 and 15.20). The set of four dihedral angles around the four axes of O–P–O–C–C of conformers **1**–**6** is classified into three types depending on the two dihedral angles around the O–P–O moiety: (1) **1** and **5**, (2) **3**, **4**, and **6**, (3) **2**.

**Figure 8:**
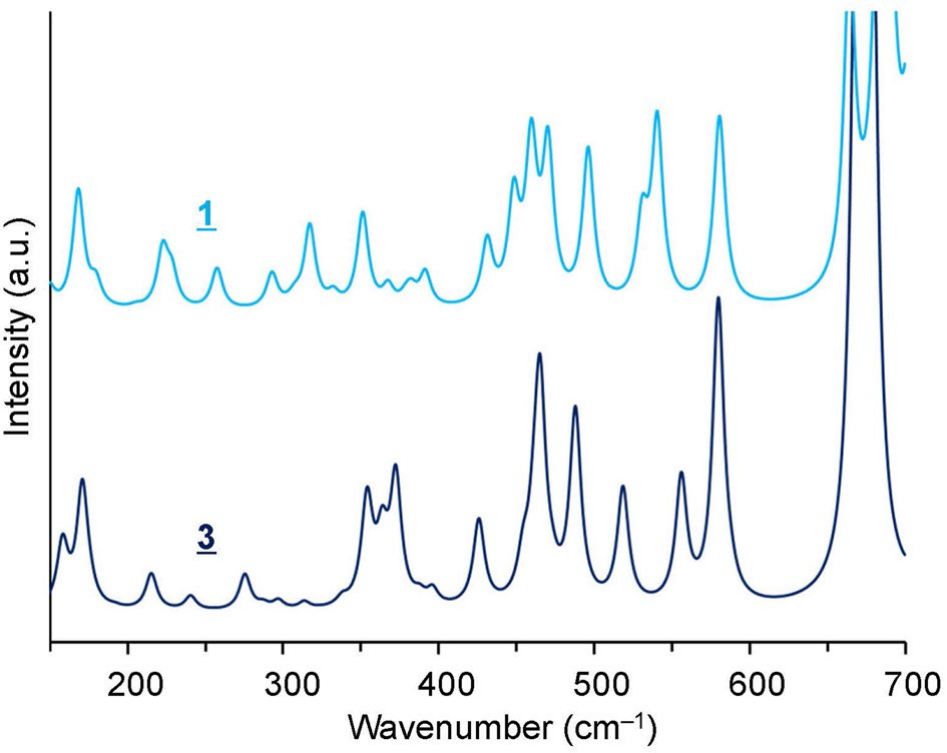
FIR spectra calculated in a solvent with a dielectric constant of *ε* = 15.20 at the B3LYP/aug-cc-pVTZ level with dispersion correction, for conformers **1** and **3** in Fig. 7. The spectra are vertically offset. The FWHM is set to the same value as that of the well-separated sharp peak at 423 cm^−1^ observed at 4 K in Fig. 2a (FWHM = 4.4 cm^−1^).

We finally chose the conformation with the best-matched calculated FIR to the observed spectrum from six conformers **1**–**6** in Fig. 7 as the MPC conformation measured here. Figure 8 shows the FIR spectra calculated in the solvent of *ε* = 15.20 for the most stable conformer **1** and the best spectral matching conformer **3** with the experimental spectrum. The number of peaks and the spectral pattern are similar between the two in the frequency region above 450 cm^−1^, and thus, frequency regions above 450 cm^−1^ appear to be insensitive to conformational differences. On the other hand, below 450 cm^−1^, the spectral patterns differ significantly between conformers **1** and **3**. In the frequency region of 350–400 cm^−1^, conformer **3** has three peaks of comparable intensity, and conformer **1** has only one strong peak. This shows that compared to conformer **1**, conformer **3** better reproduces the observed spectrum in the frequency region of 350–400 cm^−1^, as seen by comparison with Fig. 3a. We determined conformer **3** to be the MPC conformation measured in this study from the spectral pattern in the frequency region of 350–400 cm^−1^. Conformer **3** has a larger dipole moment than that of conformer **1**, as given in Fig. 7. Although conformer **3** is less stable compared to conformer **1**, conformers with larger dipole moments may tend to stabilize by orientating in the powder state. The number of peaks in the frequency region below 450 cm^−1^ of Fig. 3a means that the measured MPC is mainly in one conformation. If MPC had multiple conformations, more peaks would be observed.

## Conclusions

In order to understand the mechanism of the temperature-dependent affinity of the MPC polymers for water, temperature-dependent FIR and THz spectroscopic studies were performed on MPC, the constructing monomer for biocompatible polymers, in combination with highly accurate first-principles calculations for spectral analysis. We observed temperature-dependent spectral changes over the temperature range from cryogenic to room temperature in the low-frequency vibrations of the MPC due to the strength change or cleavage/formation of intramolecular weak hydrogen bonds at the VDW limit. The change in the experimental spectrum with temperature was interpreted by the change in the dielectric constant of the surrounding molecules using first-principles calculations incorporating the solvent effect by the polarizable continuum model (PCM). That is, it was found that the temperature-dependent spectral change with cooling is well interpreted by the change from a low dielectric constant (*ε* = 2.07) to a high dielectric constant (*ε* = 4.20). The changes in dielectric constant are the opposite of those in previously reported studies on DMAPS^19^. In the case of DMAPS, the temperature-dependent spectral changes observed with cooling are well interpreted by the change in dielectric constant from high (*ε* = 15.20) to low (*ε* = 8.33). The high dielectric constant of surrounding molecules at high temperatures is presumed to be due to the high mobility of the molecules at high temperatures. The opposite change in dielectric constant in this MPC study may be due to a change in the degree of orientation. That is, in the case of MPC, it is presumed that the orientation is more disturbed at high temperatures than at lower temperatures.

By investigating the distance of intramolecular hydrogen bonds, the spectral change was interpreted by the change in intramolecular hydrogen bond distances. In particular, it was found that the hydrogen bond of HB4 shown in Fig. 4a is cleaved at high temperature. Since HB4 is not connected to the methacryloyl group used for polymerization, the formation and cleavage of HB4 similar to monomer would occur even when polymerized. Three methyl groups bound to the nitrogen atom become more open to hydrophobic hydration by cleavage of HB4 at high temperatures, contributing to the biologically inert function of the polymerized MPC.

This report focused on temperature dependence from cryogenic to near room temperature (~ 25 °C). Since the cleavage of weak hydrogen bonds at the VDW limit occurs at approximately 100–150 K^19^, the results of this study can be extended to temperatures of approximately 37 °C that is biological and cell culture environments. Thermal excitation at ambient temperature in low-frequency modes breaks and/or reforms the hydrogen bonds^45–49^. In the temperature range from 20 to 50 °C, the probability of breaking weak hydrogen bonds is higher at the high temperature side than at the low temperature side. It is assumed that three methyl groups bound to the nitrogen atom are more open to hydrophobic hydration at the high temperature side in the temperature range from 20 to 50 °C, giving the temperature dependence observed for the hydrated state of the polymerized MPC^7^.

## Methods

### Materials

MPC was purchased from Sigma–Aldrich and measured by both synchrotron FTIR microspectroscopy and THz time-domain spectroscopy (THz-TDS) without further purification.

### Synchrotron FTIR spectroscopy

Synchrotron FTIR spectra were collected with a resolution of 2 cm^−1^ at an infrared beamline BL43IR of the SPring-8 synchrotron radiation (SR) facility (Hyogo, Japan). An FTIR (BRUKER IFS120HR) spectrometer with IR-SR as an infrared source was used. The powder sample was placed directly on a silicon substrate, inserted into the cryostat, and evacuated. By evacuation, it is estimated that the sample was free of water during the measurement, although MPC is a highly hygroscopic molecule. In fact, the absence of water was confirmed by the absence of the water libration peaks in the FIR energy region. The frequencies in Fig. 3a and the FWHM used in Fig. 3b were evaluated by fitting the spectrum with a Gaussian function. Fitting with the Lorentzian and Gaussian functions gave the same frequency and similar coefficient of determination, but the Gaussian function gave a smaller FWHM than the Lorentzian function.

### THz-TDS

THz spectra were measured with a Tochigi Nikon RT-20000 THz-TDS with a resolution of 0.8 cm^−1^. Powder samples were placed directly in the aperture region with a diameter of 2 mm on the silicon substrate and then sealed with Kapton tape. Due to the high hygroscopicity of MPC, sample setting for measurements was carefully performed in an argon gas replaced glove box under humidity control below 0.5 ppm to avoid the absorption of atmospheric water vapor into the MPC. We confirmed that Kapton tape has no absorption in the THz spectral region. The frequencies in the left panel of Fig. 5 and the FWHM used in the right panel of Fig. 5 were evaluated by fitting the spectrum with a Gaussian function. Fitting with the Lorentzian and Gaussian functions gave the same frequency and similar coefficient of determination, but the Gaussian function gave a smaller FWHM than the Lorentzian function.

### DFT calculations

To analyse the vibrational absorption spectrum of a fully amorphous powder sample, DFT calculations of gas-phase molecules surrounded by themselves were performed with the Gaussian 16 software package^50^. We utilized a hybrid Becke-type three-parameter exchange functional^51^ paired with the gradient-corrected Lee, Yang, and Parr correlation functional (B3LYP)^52,53^, and unless otherwise noted, the aug-cc-pVTZ basis set^54–58^. The Petersson-Frisch dispersion model from the APDF functional was used for dispersion correction^40^ unless otherwise noted. This model provides comparable weak interaction accuracy to CCSD(T)/aug-cc-pVTZ and can attain a consistently accurate description of VDW interactions. The geometric parameters were fully optimized, and the optimized structures were confirmed to have no imaginary frequency. The effect of the surrounding molecules was incorporated as a solvent under the PCM model using the integral equation formalism variant (IEF-PCM)^59^. To investigate the effect of various solvents and their dielectric constants, four solvent keywords accepted with SCRF = Solvent option were used. The cavity type used for PCM calculations was a scaled VDW that places a sphere with universal force field (UFF) radii^60^ scaled by a factor of 1.1 around each solute atom according to our previous paper^19^. To obtain a reliable result for very low frequencies, as in the THz region, a superfine integration grid was used. The MPC conformation was determined to obtain properly both the THz and FIR spectra from all possible conformations.

## Supplementary Information

Supplementary Information.

## Data Availability

All data generated or analysed during this study are included in this published article and its supplementary information files.
