# Supplementary material for: Van der Waals interactions regulating the hydration of 2-methacryloyloxyethyl phosphorylcholine, the constructing monomer of biocompatible polymers
Source: Sci Rep. 2022 Nov 27;12:20393. doi: 10.1038/s41598-022-24841-y (PMC9701782; doi:10.1038/s41598-022-24841-y)
Supplement: Supplementary file 1 — Supplementary Information. [file 41598_2022_24841_MOESM1_ESM.pdf]

Supplementary Information

Van der Waals interactions regulating the hydration of 2-methacryloyloxyethyl phosphorylcholine, the constructing monomer of biocompatible polymers

Masae Takahashi, Sifan Chen, Hiroshi Matsui, Nobuyuki Morimoto & Yuka Ikemoto

Contents

Table S1. Relative energies (kcal/mol) of 48 MPC conformers calculated with different dielectric constants (ε)

Figure S1. Numbers representing the 81 conformations of MPC, ABCD.

Figure S2. FIR spectra calculated at the B3LYP/6-311++G(d,p) level with dispersion correction in a solvent with dielectric constant $\varepsilon = 15.20$ for conformers **1−6** in Fig.7 in the main text. The FWHM is set to the same value as that of the well-separated sharp peak at 423 cm^{-1} observed at 4 K in Fig. 3a in the main text (FWHM = 4.4 cm^{-1}). Spectra are vertically offset.

Table S1. Relative energies (kcal/mol) of 48 MPC conformers calculated with different dielectric constants (ε)[a]

conformers[b]	Solvent-free	$\varepsilon = 8.33$	$\varepsilon = 15.20$
131Y (**1**)	0.00	0.00	0.00
3112 (**6**)	2.45	0.92	0.91
133X	2.56	1.13	–
1332 (**5**)	3.26	0.99	0.85
3X33 (**4**)	3.62	0.96	0.73
1Y11 (**2**)	4.26	0.99	0.64
1311	4.57	3.59	3.33
3311	4.60	2.78	2.49
3121	4.88	3.82	3.72
3131 (**3**)	5.35	1.30	0.65
2311	5.37	4.20	–
1133	5.44	3.88	3.59
3323	5.71	4.30	4.23
1X32	6.35	4.32	4.14
1333	6.62	2.61	2.35
1212	6.65	3.47	3.17
1111	6.73	4.41	4.23
3313	6.73	4.14	3.54
3X32	6.74	2.77	2.31
2132	6.77	4.32	–
113X	6.80	3.78	3.23
2312[c]	6.98	5.00	4.24[d]
3133	7.08	–	5.68
Y111	7.11	3.87	3.57
2X32	7.23	4.71	4.51
1323	7.44	4.62	4.47
1112	7.49	4.62	4.33
2312[c]	7.56	–	4.24[d]
1131	7.56	5.12	4.61
2313	7.60	5.53	–
1322	7.66	5.16	5.05

1123	7.75	5.58	5.46
2333	7.83	–	–
1321	8.10	5.61	5.37
1121	8.12	5.84	3.59
3333	8.15	4.93	4.55
1122	8.26	5.76	5.54
1223	8.46	–	5.53
3332	8.59	5.04	4.56
1222	8.70	–	–
3321	9.03	5.96	5.65
3123	9.04	–	–
3232	9.06	–	–
1221	9.18	6.14	5.82
3322	9.24	6.08	5.69
3221	9.41	3.76	–
3222	9.80	6.16	5.72
3223	9.90	6.25	5.78

[a]Results at the B3LYP/6-311++G(d,p) level with dispersion correction. [b]See Fig. S1 for conformational numbering. X and Y mean between 1 and 2, 2 and 3, respectively. Numbers in bold in parentheses are molecule numbers in Fig. 7 of the main text. [c]Two 2312 conformations with slight differences in the C(=O)C(CH_2)CH_3 moiety. [d]Conformation is 3X12.

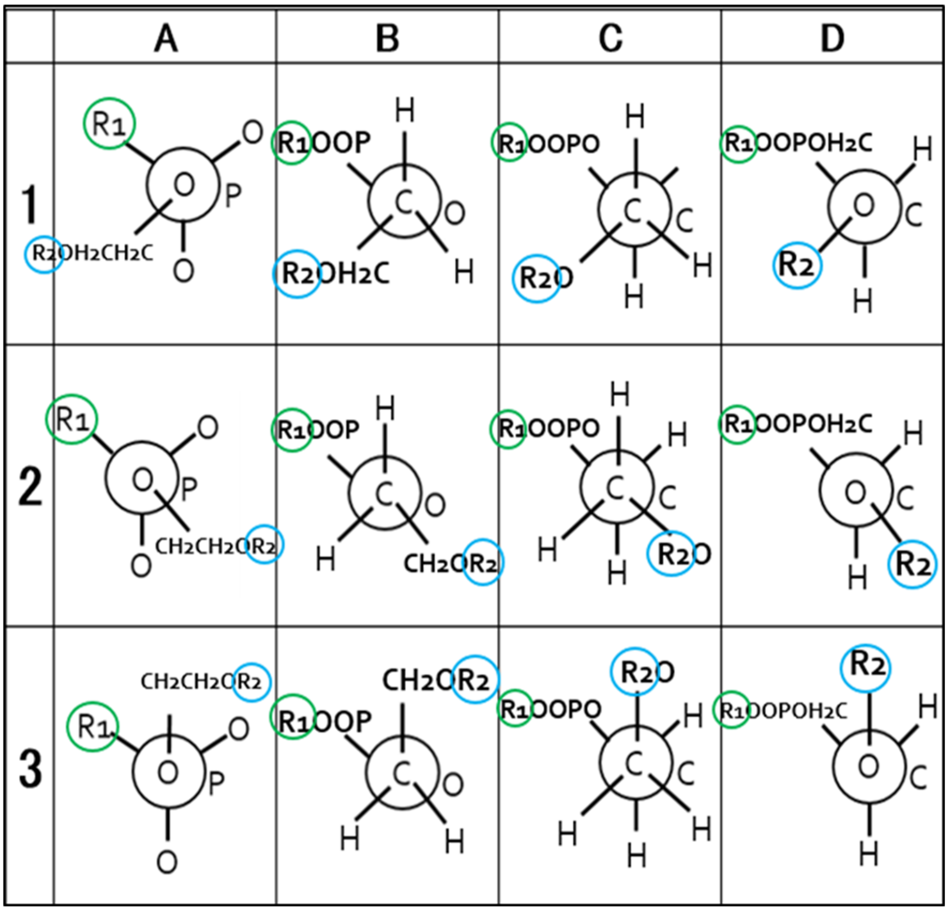

$R_1 = OCH_2CH_2N^+(CH_3)_3$, $R_2 = C(=O)C(CH_2)CH_3$

Figure S1. Numbers representing the 81 conformations of MPC, ABCD.

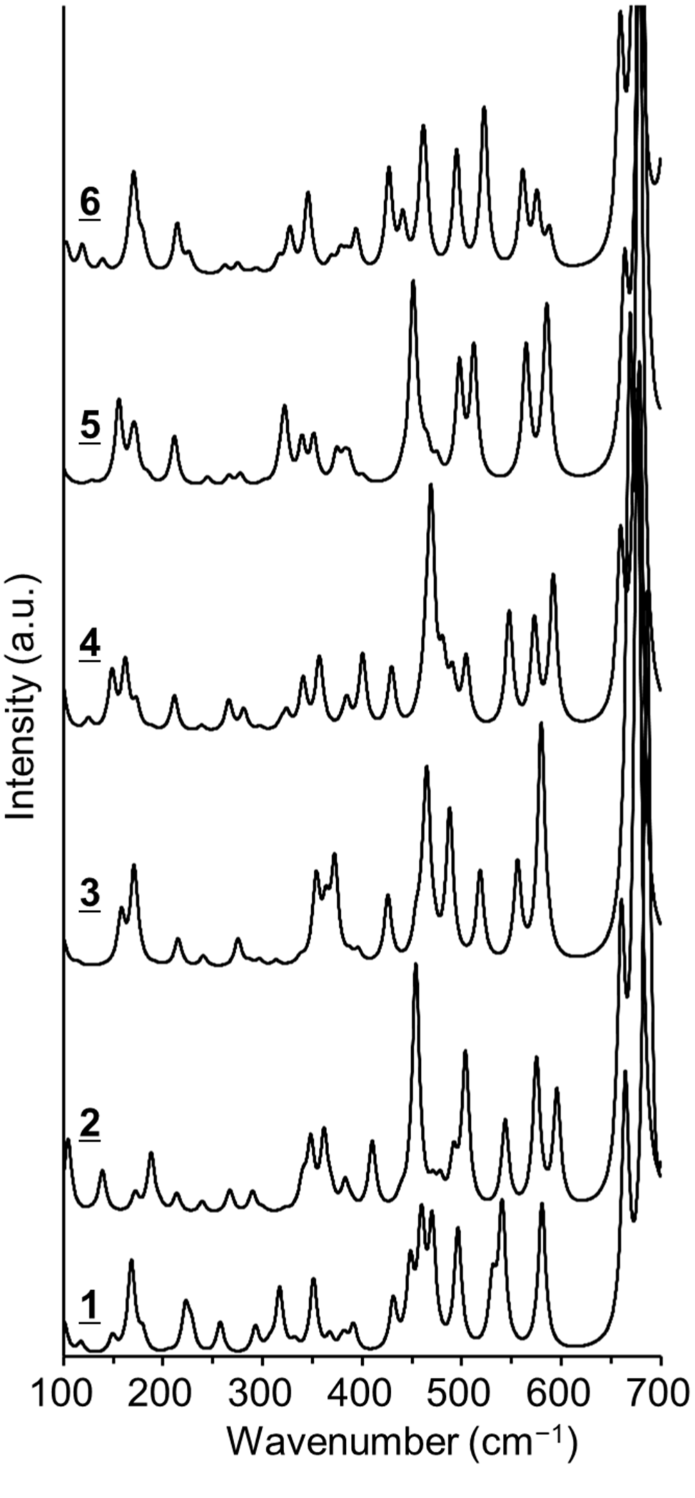

Figure S2. FIR spectra calculated at the B3LYP/6-311++G(d,p) level with dispersion correction in a solvent with dielectric constant $\varepsilon = 15.20$ for conformers **1−6** in Fig.7 in the main text. The FWHM is set to the same value as that of the well-separated sharp peak at 423 cm^{-1} observed at 4 K in Fig. 3a in the main text (FWHM = 4.4 cm^{-1}). Spectra are vertically offset.
